# AI-driven 5G IoT e-nose for whiskey classification

**DOI:** 10.1007/s10489-025-06425-1

**Published:** 2025-04-24

**Authors:** Jaume Segura-Garcia, Rafael Fayos-Jordan, Mohammad Alselek, Sergi Maicas, Miguel Arevalillo-Herraez, Enrique A. Navarro-Camba, Jose M. Alcaraz-Calero

**Affiliations:** 1 Computer Science Dpt, Universitat de València, Avda de la Universitat, s/n, Burjassot, 46100 Valencia Spain; 2 School of Computing, Engineering and Physics, University of the West of Scotland, High St, Paisley, PA1 2BE Scotland UK; 3 Microbiology and Ecology Dpt, Universitat de València, C/ Dr Moliner 50, Burjassot, 46100 Valencia Spain

**Keywords:** 5G IoT, e-nose, PCA, ML, Odor discrimination

## Abstract

The main contribution is the design, implementation and validation of a complete AI-driven electronic nose architecture to perform the classification of whiskey and acetones. This classification is of paramount important in the distillery production line of whiskey in order to predict the quality of the final product. In this work, we investigate the application of an e-nose (based on arrays of single-walled carbon nanotubes) to the distinction of two different substances, such as whiskey and acetone (as a subproduct of the distillation process), and discrimination of three different types of the same substance, such as three types of whiskies. We investigated different strategies to classify the odor data and provided a suitable approach based on random forest with accuracy of 99% and with inference times under 1.8 seconds. In the case of clearly different substances, as subproducts of the whiskey distillation process, the procedure presented achieves a high accuracy in the classification process, with an accuracy around 96%.

## Introduction

According to Forbes, “The global whiskey market size was valued at USD 62.25 billion in 2023 and it is estimated to reach USD 101.47 billion by 2032”. Along the distillation process of whiskey, there are several substances involved in the achievement of the quality of the final product, such as ethanol, water, esters, aldehydes, ketones and phenols. From them, ketones are particularly important the targeting the best possible quality of whiskey along its fermentation. Mainly, acetone predominantly appears in the foreshots or heads during a distillation process while diacetyl (2,3-butanedione, $C_4 H_6 O_2$) [1] appears in the exponential phase of fermentation. As fermentable sugars become depleted in the fermentation cycle, yeast cells metabolize these ketones. The ’ketone rest’ phase, a common practice in both breweries and distilleries, is key to reduce ketone content in the final product. They are challenging to remove during distillation and is undesirable in the final product due to its distinct buttery and slick sensory profile [2]. They can be detected at concentrations as low as 1 part per million (ppm), with higher concentrations imparting a cheesy flavor [3]. Two different approaches can be used to gain a better understanding of such ketones. On the one hand, a sensor immersed in the liquid whiskey trying to gather insights of such compounds. On the other hand, a electronic nose smelling the volatile compounds generated along the fermentation. The e-noses provided a remote-sensing capability with all the benefits associated to such kind of techniques in contract to the sensors immersed in the liquid whiskey and this is why it has been the main approach tackled in this research work.

Electronic noses (e-noses) are a device that mimics the olfactory system of animals to detect and identify odors or volatile compounds. They have a wide range of applications in various fields where the identification of such volatile compounds are of paramount importance. Likewise, they face challenges and difficulties distinguishing between complex mixtures of odors and also on detecting low concentrations of certain compounds. Thus, calibration and regular maintenance are of critical importance to ensure accuracy and reliability in such devices. Moreover, while e-noses can detect and classify odors, they do not provide direct information about the identity or structure of the compounds responsible for the odor. Despite these limitations, e-noses continue to evolve and find new applications. Ongoing research focuses on improving sensor technologies, enhancing pattern recognition algorithms, and developing portable and miniaturized e-nose devices. The have the potential to revolutionize odor analysis, quality control, and diagnostics in various industries.

It is clear the importance of understanding how whiskey smells directly affect to the final quality of the product. Thus, the main contribution of this manuscript is a complete e-nose architecture, data processing pipeline and artificial intelligence pipeline to perform the creation of unique signatures and also classification of two substances: whiskey and acetone, as the latter is an important substance in the distillation process of whiskey. And for whiskey, the system will perform as well the classification of different types of whiskeys according to their signature, being able to discriminate between single malt and blended products. The system proposed takes into account exclusively to patterns that we find in the data retrieved from the e-nose, i.e. it does not take any subjective parameters into account.

In the rest of this work, we explain the Related work. Then, an explanation of the Proposed architecture of the system, where the hardware and software are exposed. A section explaining the Inference pipeline explains the signal pre-processing, the statistical feature calculation and the application of the AI classification models. Later, a section with the Training pipeline shows the lab-based measurement procedure, the creation of the dataset, the augmentation technique applied and the AI training. The following section shows the Empirical results obtained in the whiskey discrimination process and the discrimination process of a whiskey and one of its sub-products, such as acetone. Then, the next section analyse all these results. Finally, the Conclusion section establishes a summary of the main results.

## Related work

The applications of e-noses have a wide range in different fields. In the food industry, they can be used to monitor and control the quality of food products by detecting the presence of contaminants, spoilage, or adulteration [4, 5]. In environmental monitoring, e-noses can be used to detect and identify pollutants or hazardous gases in the air or water [6–8]. They are also used in the field of healthcare for diagnosing diseases through breath analysis or detecting volatile biomarkers associated with certain medical conditions [9].

One of the advantages of electronic noses is their ability to detect odors that are imperceptible to the human nose. They can also provide fast and objective results, eliminating the subjectivity and variability associated with human sensory perception [10]. Furthermore, electronic noses can be used in environments that are hazardous or inaccessible to humans, ensuring safety and efficiency in various industries [11].

An electronic nose (e-nose) utilizes groups of sensors and algorithms to analyze the chemical composition of a sample and generate a corresponding odor profile. The basic components of an electronic nose typically include an array of chemical sensors, signal processing units, and pattern recognition algorithms. The sensor array consists of different types of chemical sensors, such as metal oxide semiconductors, conducting polymers, or quartz crystal microbalances, which respond to various volatile compounds by changing their electrical properties. When an odor or gas sample is introduced into the electronic nose, the sensors in the array detect and measure the changes in their electrical signals. These signals are then processed and analyzed by the device software and pattern recognition algorithms. By comparing the pattern obtained with a pre-established database of known odor profiles, the e-nose can identify and classify the sample based on its odor characteristics [12, 13].

In [10], the authors applied e-nose technology (with MOSES II) to differentiate between pleasant and unpleasant odors. The results were obtained with metal oxide sensors (MOx) and quartz microbalance sensors (QMB), and Machine Learning (ML) to train an artificial intelligence network (ANN) and compare the results with 123 odors (76 for training and 43 for testing), with human subjective responses obtained from a survey (in this case, odors were first individually diluted to be perceptually iso-intense), rating from “very unpleasant” to “very pleasant” in a 31 steps scale (from 0 to 30). Also in [14], the authors develop an e-nose with 7 MOx sensors, but do not use any kind of AI detection. They describe patterns for meat and other beverages.

**Fig. 1:**
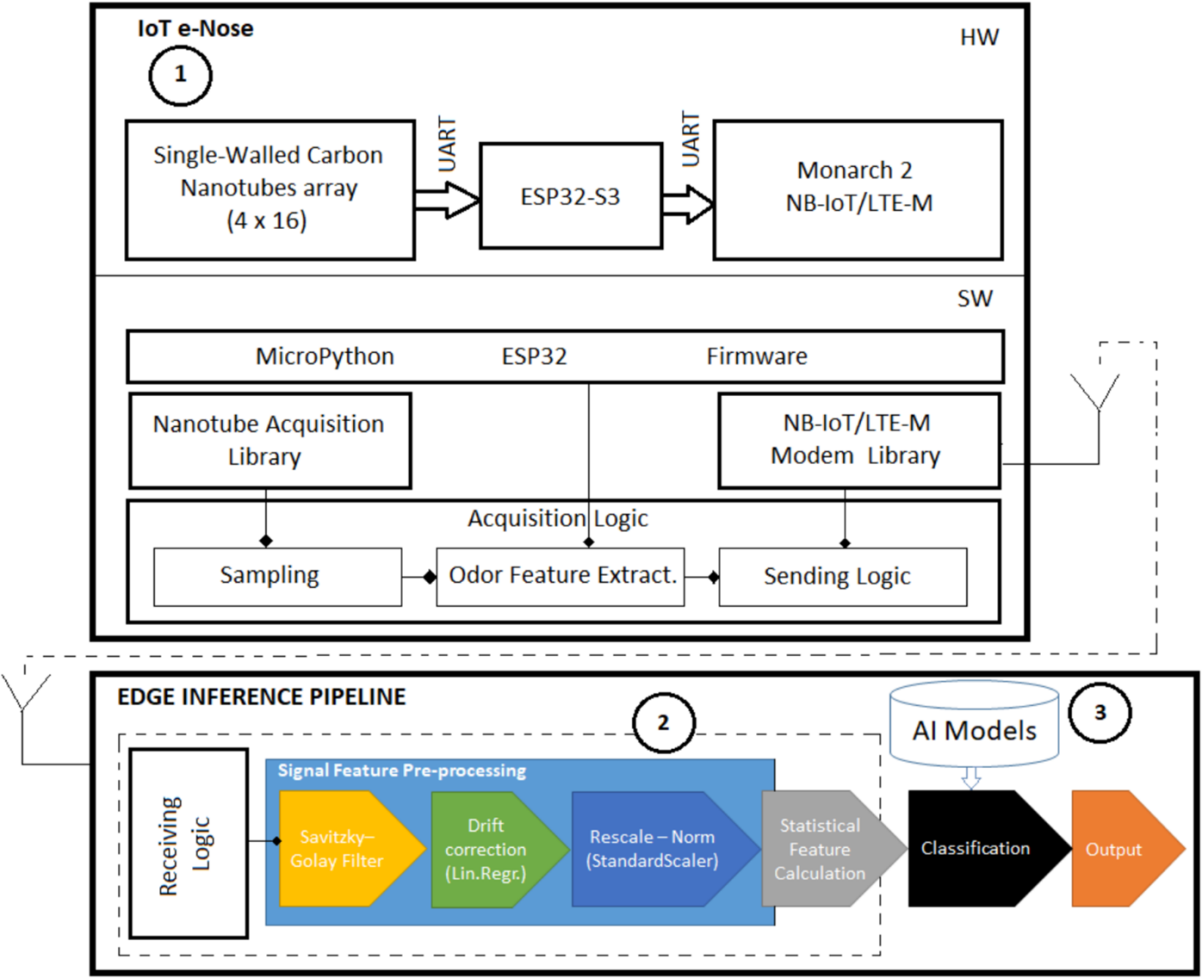
Proposed inference architecture for AI-driven e-nose Whiskey classification

In [15], the authors use a chromatography-based e-nose (HERACLES) to classify 58 different samples from 6 different distilleries. They also used a gas chromatography-quadrupole mass spectrometer (GC/MS) to determine the composing substances and a panel of experts to evaluate the subjective response.

Finally, in [16], the authors use an array of 5 MOx sensors and a set of 45 features to classify 6 types of whiskey. They use 5 ML classification methods to do three different studies: classification of whiskey brand, whiskey style identification (blended malt and single malt) and region identification. They compare their results with those of a comprehensive two-dimensional gas chromatography coupled with time-of-flight mass spectrometry device. Compared to this paper, our work differ from the previous ones in several ways. First, we focus on a complete portable hand-held IoT system suitable for on-site distillery environments, instead of complex lab-based analysis. Second, we are using a completely different detection approach, compared to previous works based on nanotube detection. Third, we are developing a complete IoT-Edge continuum system where the AI pipelines are off-loading into the Edge to provide a pervasive service. And fourth, we are providing unique signal pre-processing pipelines to handle management of smell-related signals which is a unique signature of our research work.

## Proposed architecture

Figure 1 shows an overview of the proposed architecture for AI-driven e-Nose Whiskey Classification. The figure is composed by two clearly differentiated devices. On the top part of the figure, the e-nose is the IoT device in charge of smelling and acquiring data. The details of this architecture are explained in the following subsection. On the bottom part of the figure, the reader can see an Edge point of presence where the complete data pre-processing and AI-based inference pipeline is carried out. Such pipeline will be explained in the following section.

### 5G-enabled IoT e-nose

In this work, we are using our own integrated version of the Smell-Inspector device manufactured by SmartNanotubes (https://smart-nanotubes.com/) . Consequently, we have built a micropython library for the ESP32 IoT System-on-Chip is able to interact with such a sensor. It performs continuous measurements of a 66-length vector: 64 resistance channels along with 2 channels for temperature and humidity, explained later on. The integration with SmartNanotubes e-nose makes use of a UART interface to the ESP32 [17].

Similarly, the integration with the 5G modem has been done using another UART interface connecting a Monarch 2 evaluation board kit (Monarch2-EBK) [18]. We have also embedded the micropython Monarch 2 library to allow 5G-enabled wireless low-power IoT communications.

**Table 1:** Mapping between e-nose sensors and the 15 odor features

Detect.	S1	S2	S3	S4	S5	S6	S7	S8	S9	S10	S11	S12	S13	S14	S15	S16
Array 1	REF	REF	$f_{11}$	$f_{11}$	$f_{11}$	$f_{3}$	$f_{3}$	$f_{3}$	$f_{2}$	$f_{2}$	$f_{2}$	$f_{1}$	$f_{1}$	$f_{1}$	REF	REF
Array 2	REF	REF	REF	REF	REF	$f_{9}$	$f_{9}$	$f_{9}$	$f_{6}$	$f_{6}$	$f_{6}$	$f_{5}$	$f_{5}$	$f_{5}$	REF	REF
Array 3	REF	REF	$f_{14}$	$f_{14}$	$f_{14}$	$f_{10}$	$f_{10}$	$f_{10}$	$f_{7}$	$f_{7}$	$f_{7}$	$f_{4}$	$f_{4}$	$f_{4}$	REF	REF
Array 4	REF	REF	$f_{15}$	$f_{15}$	$f_{15}$	$f_{13}$	$f_{13}$	$f_{13}$	$f_{12}$	$f_{12}$	$f_{12}$	$f_{8}$	$f_{8}$	$f_{8}$	REF	REF

The acquisition logic is straight-forward as it is a continuous loop smelling, performing the odor feature extraction, explained in the following subsection and sending data via 5G modem to the pipeline deployed in the Edge.

### Odor feature extraction

The e-nose device consists of 4 arrays of 16 sensors. Each sensor consists of a resistor and a Single-Walled Carbon Nanotube (sw-CNTs) [19]. When volatile compounds come into contact with the sw-CNTs, they induce changes in their resistance. This resistance alteration is translated into an electrical signal, which is then converted into a numerical output. This process occurs at intervals of 1.8 seconds. Each one of the 64 sensors collaborates to smell a set of 15 odor features $\{f_1\ldots f_{15}\}$. Table 1 shows what sensors contribute to each feature. In this table, REF indicates special sensors that are used as references for calibration purposes.

To obtain the values for each odor feature, the values read by all sensors related to that feature are averaged, according to the manufacturer’s specification [20]. For example, the value for feature $f_3$ is obtained by averaging the values reported by the sensors S6, S7, and S8 in the first array.

Type 1, 3 and 4 arrays have the same number of channels and their behaviors are established by construction as described in [19], where each channel is composed of a gold cell with a gap between the two terminals on a silicon dioxide substrate. Between these two terminals we find a carbon nanotube that determines the resistance characteristics of the cell. According to this resistance characteristic, each channel has a different behaviour and these are grouped as described in Table 1. In addition, the Type 2 array is different by construction as it has more reference channels. We have taken this into account when sampling and making time average with the e-nose.

**Fig. 2:**
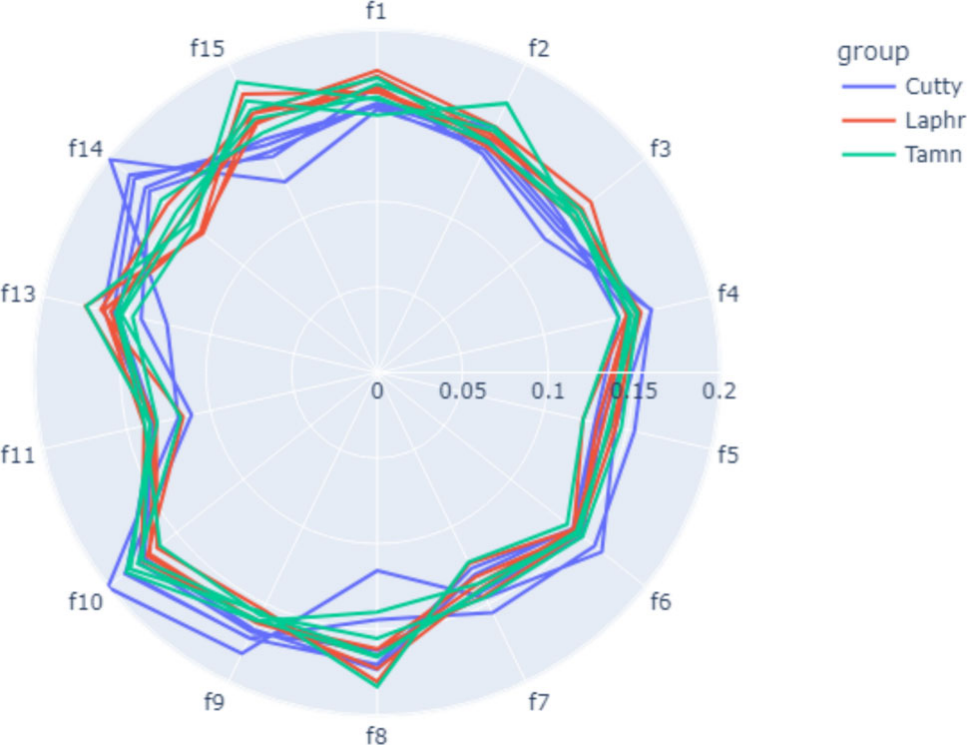
Radial plot of the 15 features for 3 different whiskey brands (5 measurements each)

**Fig. 3:**
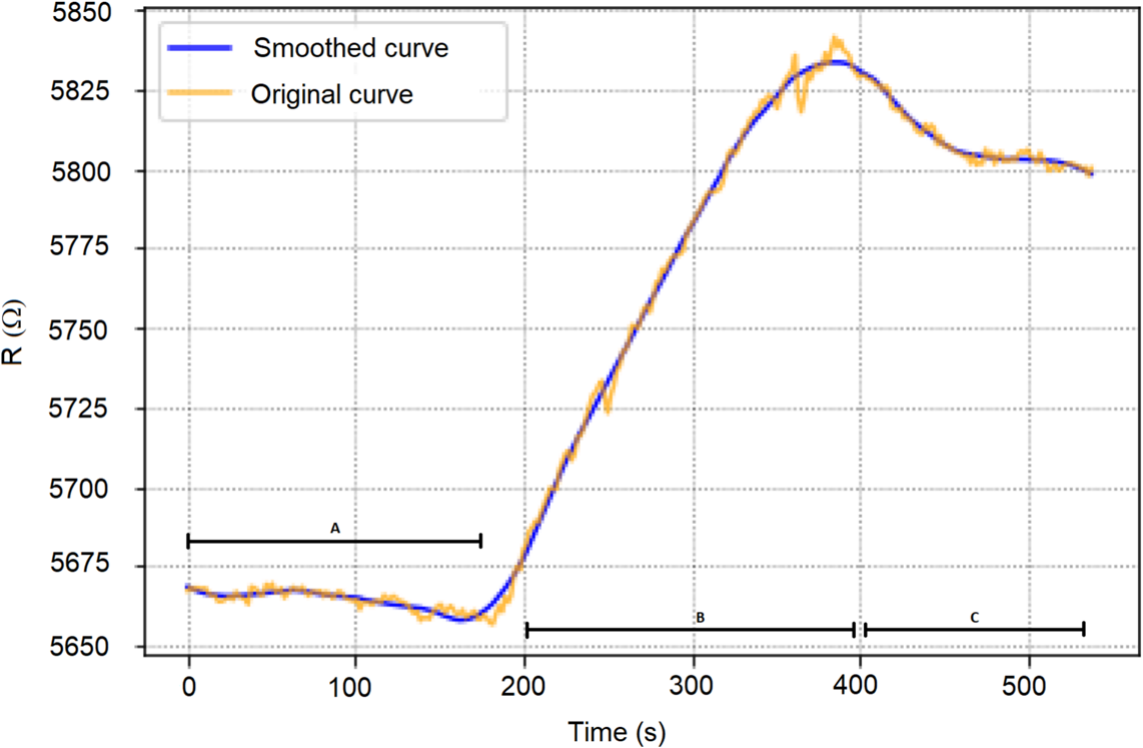
Original vs. smoothed signal comparison. Savitzy-Golay filter applied

Figure 2 shows a radial plot representing some samples of signature of smell measurement for different whiskey brands. Such signature is composed by the drawing of such 15 different smell features previous mentioned. At a glance, the reader can see how there are small differences among substances but also the complexity associated to the classification problem as there are a lot of similarities, overlapping, and even variations between measurements of the same product. This represent a challenging pre-processing stage where the dataset is prepared to be fruitful and efficient for AI-driven classification tasks. Such AI pipeline processing is explained in the following section.

## Inference pipeline for whiskey classification

The bottom part of Fig. 1 shows the overall steps involved in the AI pipeline that has been used for the measurements, feature extraction and classification. The following subsections are explaining in detail step of the consecutive steps designed in the pipeline.

### Noise reduction filter

This step is represented as Savitzky-Golay Filter in the Edge Inference Pipeline part in Fig. 1. Figure 3 shows as a running example a concrete sample of the original signal acquired associated to a concrete feature, f2 in yellow colour. The values plotted are already the average values of the different channels measuring such feature. Notice that despite of such averaging, the signal still presenting clearly noise along the complete exposition time line. This noise will cause significant degradation in the accuracy of the creation of unique signatures for each of the different substances. This is why we propose to apply a Savitzky-Golay smoothing filter [21], defined by function *F* in order to avoid abrupt variations and smooth extreme values. Such filter takes into consideration a window of values in order to apply the smoothing of the a given sample and replace such values by a N-degree polynomial interpolation of such values, thus performing the smoothing of the signal. In our case, the following parameters have been decided based on an empirical try-and-optimize approach until we gather the best trade-off between latency budget and accuracy. Specifically, we conducted an automated grid search, varying window sizes from 1 to 100 samples and polynomial degrees from 1 to 10. This tuning process utilized a reduced subset of samples specifically captured for this purpose. To ensure practical applicability, we imposed a maximum computation time limit of 100 milliseconds (which is 50 milliseconds (which is around 3% of the sampling time), effectively constraining solutions that might result in excessive latency. The resulting parameters were a window-size of 51 points and 3-degree polynomial interpolation.

1 $$\begin{aligned} S_{smooth} = F_{51,3}(S_{raw}) \end{aligned}$$

The results of the application of such filter is also depicted over the same running example in Fig. 3 to allow the reader to understand the effect of such function over the original signal.

### Drift correction

At this stage, the reader needs to know that our measurement methodology is split in three steps. A preparation step where the device is not exposed to the substance, a exposition step where the device is smelling the substance and a post-exposition step where the device is again not smelling the substance. Using the running example depicted in Fig. 3, the steps are labelled with letters: A, B and C, respectively. In the rest of the manuscript, authors will refer to non-exposition phase defined as steps A and C and to exposition phase defined as step B.

Now, if the reader focuses on analysing each of the three steps previously introduced in the running example of Fig. 3, it will soon realize how the signal is continuously decreasing along the time within each of the steps, mainly due to the long-exposition of the substance to the nano tubes causes certain resistance against the continuous stimulation of resistors. This is why it has been decided to straighten the signal up. This steps is represented as Drift Correction in Fig. 1. The process is as follows.

**Fig. 4:**
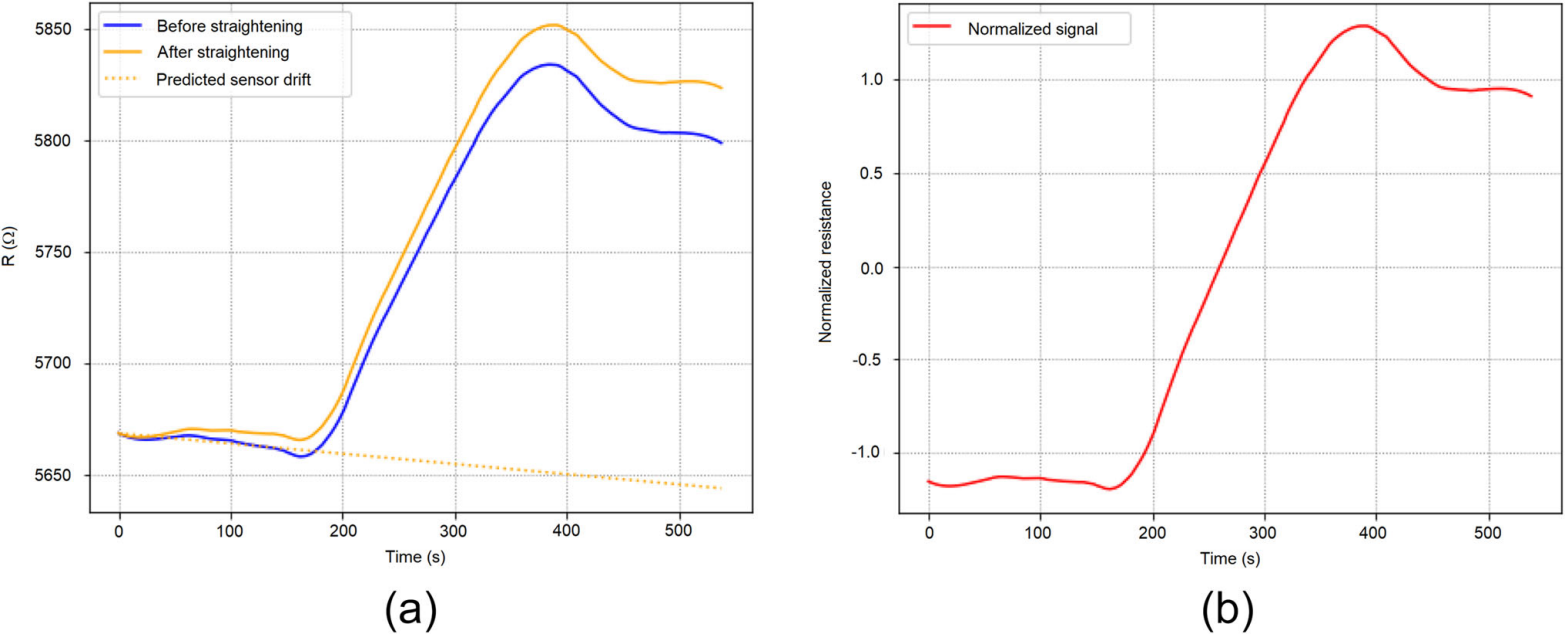
Signal drifting pre-processing steps (a) and signal normalization step (b)

**Table 2:** List of statistical features used for classification

Indicator	Description	ID
*R*	The maximum value of a signal	*R*
	over the second phase (exposition).	
$R_0$	The mean of a signal over	$R_0$
	the first phase (non-exposition or air).	
$R_{10}$, $R_{25}$,	The means of the signal at 10%, 25%, 50%, 75%	$R_{10}$, $R_{25}$,
$R_{50}$ and $R_{75}$	of the time of the exposition phase (i.e. steps B).	$R_{50}$ and $R_{75}$
$R - R_0$	Difference between the exposition	$Ind_1$ and
	non-exposition phase (i.e. steps A and C)	
$\frac{R - R_0}{R_0}$	Difference between the exposition and	$Ind_2$
	non-exposition phase but scaled by the baseline value.	
$\frac{\frac{R - R_0}{R_0}}{\sqrt{\sum _{i=0}^{N}{( \frac{R_{0i}-R_i}{R_0} )}^2}}$	Recommended indicators for this task (N=300).	$Ind_3$ [24]

First, for each of the different features previously smoothed, it is estimated the signal drift due to the ambient air by performing a linear regression over the phase of exposition to normal air, i.e. no exposition to the substance . Using the result of such linear regression, we predict the drift of the sensor through the whole signal and we perform the subtract of such drift from the smoothed signal. At the end, we added the intercept of the linear regression to readjust the signal (Fig. 4). This is formally defined as:

2 $$\begin{aligned} S_{drifted} = S_{smooth} - D_p + S_{smooth}^0 \end{aligned}$$

Equation 2 shows the formula designed to calculate the drifted signal drifted. Let us define: $S_{smooth}$ as the smoothed signal, $D_p$ as the predicted drift of the sensor using a linear regression over 100 samples (i.e. the first step of the non-exposition phase), and $S_{smooth}^0$ as the first value of S.

Figure 4-a shows the smooth signal calculated in previous step and the result of the drifting of such signal using the (2). The reader can notice now how the decay along the time has been mitigated and the signal is more much consistent and clean along different expositions to the substance. The same Fig. 4-a shows as well the plot for the calculated linear drifting (i,e. the value of $D_p$).

### Normalization

The next step is to rescale the signal to be able to have comparable measurements and focus on the shape of each signal. This steps is represented as Rescale - Norm in Fig. 1. To do this, we used Scikit-Learn’s StandardScaler [22, 23] which consists in center-reducing applied over the signal. This is formally defined as in (3).

3 $$\begin{aligned} S_{norm} =\frac{S_{drifted} - \mu }{\sigma } \end{aligned}$$

Equation 3 shows the rescaled formula where let define: $S_{drifted}$ as the drifted signal with a length of 300 samples, i.e. includes all the steps and phases, $\mu$ as the mean of $S_{drifted}$, and $\sigma$ as the standard error of $S_{drifted}$.

The result of this rescaling is shown in the Fig. 4-b for the same running example being used along the manuscript.

### Statistical feature calculation

In order to provide the classification tasks with high quality descriptive information, it has been decided to calculate the following indicator that are later on used as input to the classification task. The idea behind is to extract indicators that describe the signals’ specificities, to allow the classifiers to find differences between each one of them. Table 2 shows the definition and detailed description of the different statistical features used for the classification of the different odors.

**Fig. 5:**
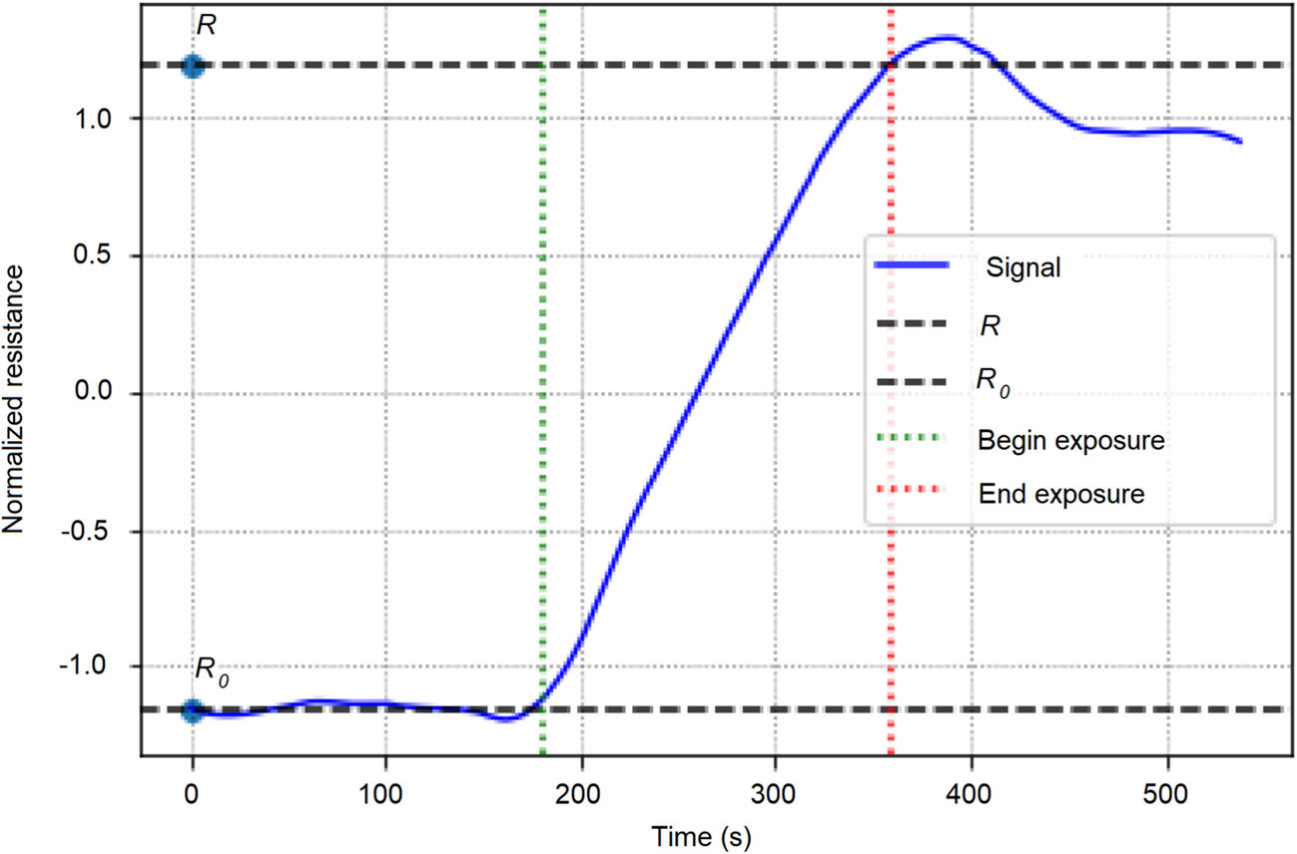
Acquisition phases. Visualization of *R* and $R_0$ in a signal

Figure 5 depicts both *R* and $R_0$, as defined in Table 2, for the same running example used along the manuscript. It is worth describing the global shape of the signal. After the end of the exposition phase, the signal curve does not get back to its initial value, this is because the sensor gets saturated with the volatile compounds and it takes time for it to get back to its initial value. Moreover, we observed that the more measurements we made during the day and the less sensitive the sensor got. In fact, at some point, the sensor gets too much saturated and making measurements is almost pointless because the device barely reacts to odors. Also, just before the beginning of the exposure, we can observe a slight decrease in the resistance which could be related to the movement we make when we put the device on the bottle of the compound.

A significant analysis has been carried out in order to determine what is the most efficient design of the input of the AI model. The size of the input to the AI model is fixed and it is always equals to 15, 1 per each of the different features smelled. However, notice that a raw value can be inserted to the AI model or an statistical features can be inserted. This is why all the statistical features available in Table 2 has been empirically used as input to the AI models in order to analysis which one produce better results. See results section to see the description of the findings.

### AI classification models

It has been decided to allow the usage of different machine-learning techniques into the inference pipeline to compare them for the job of classifying whiskies and acetone. In principle the pipeline is extensible and thus allows to plug any AI model. However, in our prototypes we have validated the following ones: Gaussian Naive Bayes, Random Forests, Logistic Regression, Support Vector Machine (SVM) classifier and a Multi-layer perceptron Neural Network (MLP).

We used the Gaussian Naive Bayes model because this model is known to be efficient on small datasets like ours. We have performed a logistic regression using the *lbfgs* solver (limited-memory Broyden-Fletcher-Goldfarb-Shanno’s algorithm). The Random Forest technique is run using the Gini criterion (as it is much faster, because it is less computationally expensive than other criteria, such as decision tree with Entropy criterion). The Neural Network used is a multi-perception (fully-connected) which contains one hidden layer of 5 neurons using the *relu* activation function and *lbfgs* for weight optimization. We also have performed a Support Vector Machine (SVM) classifier. We used the models provided by $scikit-learn$ for each one of those models. Even not having to worry much about computational complexity as we are running the inference pipeline in the Edge, we have decided to keep as minimal and simple as possible the AI models with the intention in the future to explore as well in-device IoT pipelines, this is why we have not explored any Deep Neural Network.

As a result, the classifier provides the detection of whiskey and acetone and also the discrimination of the different types of whiskeys being detected.

**Fig. 6:**
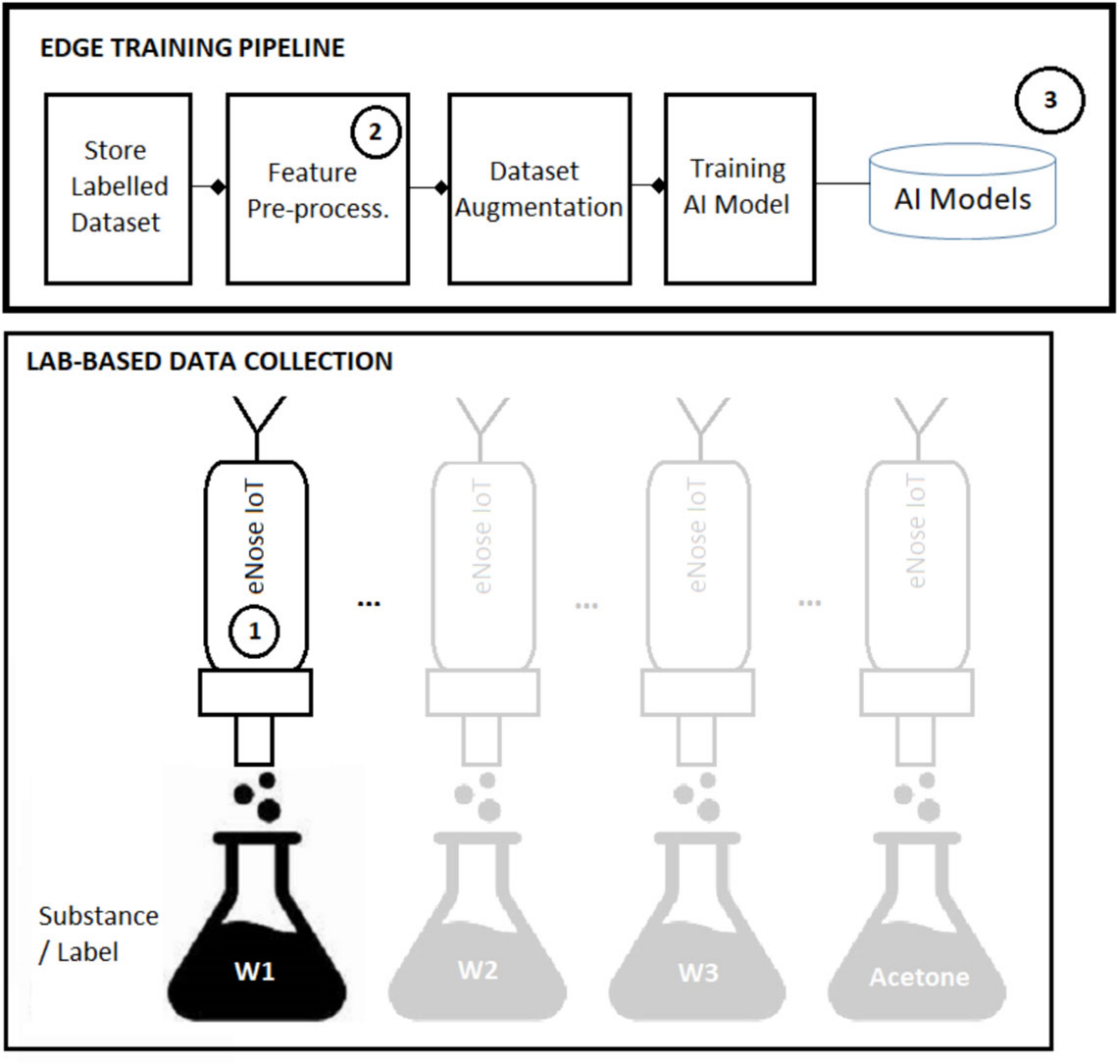
Training pipeline architecture for Whiskey classification

**Fig. 7:**
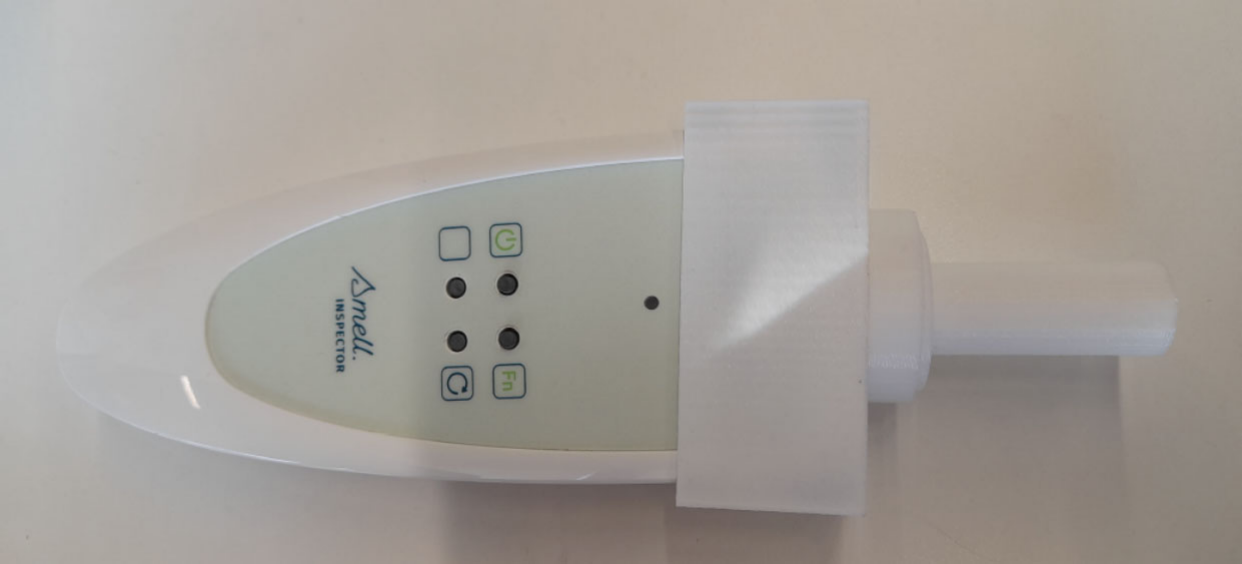
Adaptator for the e-nose

The training pipeline of each of these different AI models is explained in the following section.

## Training pipeline

Figure 6 shows the training pipeline used to perform the creation of the dataset and AI models being used later on the inference pipeline. First, the data is measured using the same device that will be used for the inference pipeline and labelled in a lab environment and stored as dataset. See the device in Fig. 6 is labelled with number 1 and how it matches with the architecture presented in Fig. 1 under the labelled with the same number. Second, the same pre-processing pipeline is carried out over the data set in order to generate the inputs to the AI models. See again how the labelled with number 2 in Fig. 6 represented the same sub-pipeline depicted in the labelled with number 2 in Fig. 1. After that, and mainly due to the fact of dealing with small datasets, a Dataset augmentation technique has been carried and finally the training of the AI models has been performed using such augmented dataset. As a result of such training the trained AI models are generated, see labelled with number 3 in Fig. 6. These AI models are exactly those being used in the interference pipeline as labelled with number 3 in Fig. 1. The following subsections describe different aspects related to the training phase of the AI models.

### Lab environment

It has been created a lab-based data collection environment, as sketched in the bottom part of Fig. 6. In order to standardize the spatial conditions of every measurement, we designed and printed a receptacle for the e-nose with a 3D printer. This receptable is used with the intention to minimize any odour interference between our measures so that it was printed a specific adaptator for each bottle of substance so that the odours do not mix, this is shown in Fig. 7. This receptable allows the e-nose to always have the same position when doing the measurements and also allows to perform the measurements directly from the bottles. In fact, manipulation involving the whiskey being fully exposed to air and then put back into the bottle can alter the quality of the whiskey (although due to the high alcohol content, whiskies are much more stable than other fermented beverages and do not deteriorate due to microbiological spoilage [25]), as oxidation or contamination processes can be produced.

**Table 3:** List of hyperparameters employed in the models used for classification

Model	Default hyperparameters
Gaussian Naive Bayes	priors=None, var_smoothing=1e-09
Logistic Regress.	penalty=’l2’, dual=False, tol=0.0001, C=1.0, fit_intercept=True, intercept_scaling=1,
	class_weight=None, random_state=None, solver=’lbfgs’, max_iter=100,
	verbose=0, warm_start=False, n_jobs=None, l1_ratio=None
Random forest classifier	n_estimators=100, criterion=’gini’, max_depth=None, min_samples_split=2,
	min_samples_leaf=1, min_weight_fraction_leaf=0.0, max_features=’sqrt’,
	max_leaf_nodes=None, min_impurity_decrease=0.0, bootstrap=True, oob_score=False,
	n_jobs=None, random_state=None, verbose=0, warm_start=False, class_weight=None,
	ccp_alpha=0.0, max_samples=None, monotonic_cst=None
MLP Classifier	hidden_layer_sizes=(100,), activation=’relu’, solver=’adam’, alpha=0.0001,
	batch_size=’auto’, learning_rate=’constant’, learning_rate_init=0.001, power_t=0.5,
	max_iter=200, shuffle=True, random_state=None, tol=0.0001, verbose=False,
	warm_start=False, momentum=0.9, nesterovs_momentum=True, early_stopping=False,
	validation_fraction=0.1, beta_1=0.9, beta_2=0.999, epsilon=1e-08,
	n_iter_no_change=10, max_fun=15000
SVM Classifier	C=1.0, kernel=’rbf’,degree=3, gamma=’scale’, coef0=0.0, shrinking=True,
	probability=False, tol=0.001, cache_size=200, class_weight=None, verbose=False,
	max_iter=-1, decision_function_shape=’ovr’, break_ties=False, random_state=None

### Dataset

The 4 substances smelled has concretely been: one single malt speyside whiskey (T - Tamnavulin), one blended malt and fruity whiskey (C - Cutty Shark Prohibited Edition), one single malt and peaty or smoky whiskey (L - Laphroaig), one acetone at 100% purity. For each of them, 5 different measurement campaigns have been carried out, each one is a different day, in summer. We used different humidity and temperature conditions as they were made on different times of the day. In every campaign, it has been gathered 300 samples of each of the substances. These 300 samples are equally distributed along the 3 steps involved in the sampling: exposition to normal air, exposition to the odor and exposition to normal air, having 100 samples for each of these steps. It is worth mentioning that every sample is gathered in average every 1.8 seconds, thus it rooks around 540 seconds on gathering the measurements of every substance. At the end of the measurement, the total dataset obtained is composed by 6000 labelled samples where 2000 are in fact corresponding to the exposition phase.

Between each measurement, we waited at least 20 minutes for the e-nose to stabilize again on normal air. Ideally, we should have always waited for the sensor to be perfectly stable but in real conditions, the sensor always has a drift and we decided to tolerate a reasonable amount of sensor drift that would be corrected later in the process.

After the aforementioned measurement process, the processed records are stored in csv files in the Edge. These records register also the label of the smelled substance.

### Data augmentation

When the samples maybe not enough to achieve statistical power, the dataset can be augmented using the Synthetic Minority Oversampling Technique (SMOTE) [26] . This technique can be applied in situations where datasets have very few examples of a given class, such as in rare disease diagnosis or fraudulent transactions, training models can result in poor performance due to the imbalance. Traditional methods to address this issue include under-sampling the majority class, which can lead to loss of valuable data and increased bias, or over-sampling the minority class, which can cause overfitting. SMOTE offers a solution by generating synthetic examples for the minority classes. This can be done by calculating the difference between a sample and its nearest neighbor, multiplying this difference by a random number, and adding it to the sample to create new, synthetic data points, thus improving the balance without overfitting. Notice that our dataset is imbalanced by design as it has the double of samples in the non-exposition phase than in the exposition phase. This is why SMOTE has been used to generate an additional 100% of synthetic samples associated to the exposition phase, i.e. 2000 samples (500 of each substance), leading to a total of 8000 samples. SMOTE makes use of K-neighbours in order to perform the generation of results. It has been decided to make use of 4-neighbours in order to make sure we are able to capture the highest entropy where the number of neighbours is completely heterogeneous, i.e. one belongs to each class. Thus, since we are dealing with 4 different classes, this number of neighbour been decided to match it.

### AI training

Each of the AI models being trained has associated different hyper-parameters [23]. Table 3 summarizes them with their default options:

## Empirical results

### Whiskey discrimination accuracy results

For each AI model trained, it has been performed a 5 K-fold cross-validation on our dataset and retrieved the mean of the accuracy achieved. The specification of an algorithm to distinguish between different types of whiskey will help to establish rules for the categorization of a unique substance with different characteristics.

**Fig. 8:**
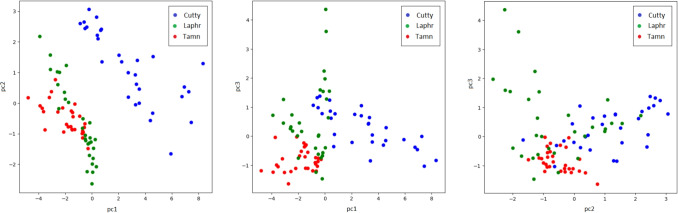
PCA analysis for the three components of the three whiskies dataset after SMOTE

The analysis of the principal component analysis (PCA), shown in Fig. 8, is a 2D graph for the 3 components since there are 3 different types of whiskey being analyzed. It shows a clear separation for blended whiskey from others, but it is not as clear as in the case of single malt whiskies. The comparative analysis between different AI methods and the statistical features that are used as input to such methods, shown in Table 4 is very insightful.

On the one hand, it is worth indicating that the usage of the first four statistical features as an added value is the fact that only a subset of the sampling (10%, 24%, 50%, and 75%) is needed to perform the classification, while the last four statistical features need the complete set of samples, i.e. 100%. This is useful when analysis the trade-off between latency budget and accuracy. From this analysis, it is worth mentioning that the best indicator is *R*. On the other hand, the best AI model performing that task is Random Forest (RF) classifier as the best method to perform the discrimination of whiskeys. When we combine both, the accuracy achieved is 99%, using RF classifier and R as an input of this classifier. Here, the different methods selected have specificities within the context and characteristics of the selected dataset. With this dataset, Naive Bayes assumes feature independence, which might not hold for whiskey data where features could be highly correlated. In the case of the Logistic Regression, it is limited to linear decision boundaries, which might not suffice for capturing complex feature interactions in whiskey classification. For an MLP Classifier (Neural Network), it might require more data to achieve optimal performance and can overfit small datasets. In the case of SVM, it would be effective for binary classification and specific kernels but can struggle with multiclass problems or require extensive parameter tuning. Taking into account the previous explanations, in comparison, the Random Forest’s ability to model complex, nonlinear relationships, robustness to noise, and resistance to overfitting likely explain its better performance in our whiskey classification task.

**Table 4:** Accuracy comparison for whiskies classification models

Models	$R_{10}$	$R_{25}$	$R_{50}$	$R_{75}$	*R*	$Ind_1$	$Ind_2$	$Ind_3$	Average
Naive Bayes	0.56	0.70	0.37	0.37	0.99	0.93	0.93	0.99	0.73
Logistic regress.	0.56	0.52	0.37	0.22	0.89	0.70	0.82	0.78	0.61
Random forest	0.59	0.48	0.78	0.48	0.99	0.99	0.99	0.99	**0.79**
MLP Classifier	0.52	0.52	0.48	0.52	0.99	0.82	0.93	0.82	0.70
SVM Classifier	0.56	0.59	0.41	0.19	0.89	0.70	0.82	0.82	0.62
Average	0.56	0.56	0.48	0.36	**0.95**	0.83	0.90	0.88	

Bold refers to the best result

**Fig. 9:**
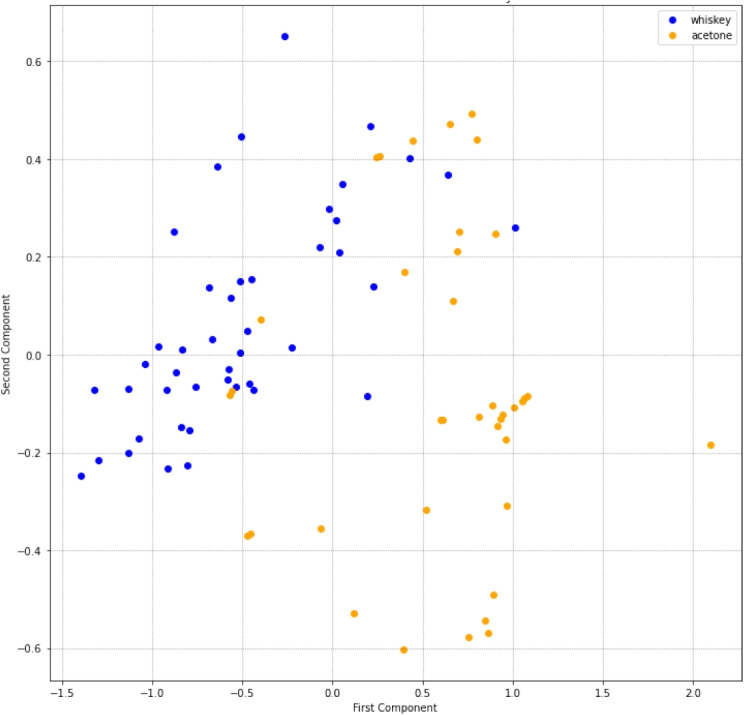
PCA analysis of our dataset for acetone and whiskey substances after SMOTE

### Acetone and whiskey discrimination results

Analogously, the PCA in Fig. 9 depicts a clear separation between the two substances. is a 2D graph as there are only 2 different substances being analyzed.

Table 5 shows as well an analogous analysis but this time related to the discrimination between acetone and whiskeys. From this analysis, it is worth mentioning that surprisingly the best indicator is $R_{10}$ which is in fact the one that requires less latency budget converting it in the best choice. With respect to the best AI model to perform the task, MLP classifier is clearly the best method to perform the discrimination of whiskeys. When, combining both of them, it is achieved an accuracy of 96% using MLP classifier and $R_{10}$ into such classifier.

**Table 5:** Accuracy comparison for classification models to differentiate between two substances

Models	$R_{10}$	$R_{25}$	$R_{50}$	$R_{75}$	*R*	$Ind_1$	$Ind_2$	$Ind_3$	Average
Naive Bayes	0.86	0.83	0.83	0.79	0.82	0.84	0.81	0.89	0.83
Logistic regress.	0.89	0.86	0.82	0.77	0.78	0.86	0.70	0.60	0.79
Random forests	0.94	0.86	0.85	0.79	0.83	0.84	0.83	0.91	0.86
MLP Classifier	0.96	0.98	0.90	0.92	0.93	0.95	0.86	0.73	**0.90**
SVM Classifier	0.86	0.87	0.72	0.72	0.67	0.82	0.51	0.48	0.71
Average	**0.90**	0.88	0.82	0.80	0.81	0.86	0.74	0.72	

Bold refers to the best result

### Inference and training times

With respect to the inference time, it is worth mentioning that the system needs to warm up until it can go for continuous monitoring in real time of the substances. The time for warming up is 91.3s, which is the time required to measure at least 51 measurements as this is the requirement to perform the data pre-processing stage of smoothing. As this is working with a sliding window, after such filling of the buffers, the system will infer at constant speed. The inference time for the execution of the best AI models for whiskey discrimination and the best AI model for whiskey and acetone discrimination is exactly the same as the difference in nanosecond scale, they are exactly: 1.5 seconds related to the data acquisition, sent, reception and pre-processing and 0.02s for the inference time. It is important to mention that this inference processing time is perfectly suitable as a new measurement is performed every 1.8 seconds, and thus it allows to keep constant pace between sampling and processing, even it has room to send the device to sleep for 0.3 seconds.

With respect to training times, i.e., the time required to perform the training of the AI models when they are trained with the dataset previously described is indicated in Table 6. It is worth noticing the capability of performing almost instant training which suggests the possibility to explore any on-line adaptive training pipeline in the future as the longest process takes 0.6 to perform the complete training from scratch.

**Table 6:** List of training times for the models used in the classification

Model	Training time
Naive Bayes	0.007s
Logistic regress.	0.629s
Random forests	0.024s
MLP Classifier	0.413s
SVM Classifier	$<10^{-3}$s

## Results analysis

The results obtained using this entire architecture presented demonstrate that machine learning can be applied to odor recognition using the Smell Inspector device. The best statistical features suitable for the classification between two substances such as whiskey and acetone in this work were the indicator $R_{10}$. That could be explained by the extreme volatility of acetone that might have allowed the substance to reach the sensors faster than whiskies during the first 10% of each measure. For the case of the classification between 3 types of the same substance, such as three brands of whiskies, the best performance indicator was *R* on average.

The initial objective of this project was to classify and distinguish different types of whiskies (for blind-tasting purposes). We have achieved separability in classifying them properly, but some of the characteristics of these fermented beverages are difficult to discriminate because they are quite close in terms of Euclidian distance in the 3 dimension PCA space. For this reason, we decided to distinguish between two different substances, one of these whiskies and a sub-product of distillation like acetone. In fact, even if whiskies smell a bit differently, they have a similar composition when they have a similar process of production (this is the case for the single malt distillation process), but the blending process could allow further differences. That could explain why it is difficult to separate them.

In terms of external conditions, the temperature and humidity were taken into account (the measurements were made in summer and these characteristics were in the range of 27 to 32 $^{\circ }$ C and 28- 32% of air humidity), but we might also think about two other things: the sensor saturation and its drift. In fact, even if we correct the drift in our process, the method is not perfect as the drift is not perfectly linear. The saturation happens when the sensor has already been working for a while and loses its sensitivity to the substances.

**Table 7:** Accuracy obtained for the AI models with the pre-processed data

Models	Accuracy with raw normalized data
Naive Bayes	0.704
Logistic regress.	0.630
Random forest	0.815
MLP Classifier	0.704
SVM Classifier	0.593

It is worth mentioning that previous work [15, 16] addressing the discrimination of whiskey brands have based the design of the inputs to their AI models on raw normalized data. This is the equivalent to directly insert the data after it has been pre-processed into the AI models rather than creating the statistical features being designed in our contribution. Table 7 shows the accuracy of the AI models using directly the pre-processed signal as input in order to allow the reader to see how the presented method based on statistical features provides better results for all the case. For example, for Random Foster we gather an accuracy of 81% far below the 99% achieved with the *R* statistical feature. This is a significant advancement to the state of the art.

## Conclusions

A complete architecture and system has been designed, prototyped and validation to perform the effective discrimination of different whiskeys and acetone using the e-nose Smell Inspector from SmartNanotubes. We analysed in detail the signals received by e-noses and perform a tailored signal processing pipeline to clean and prepare the signal for AI-driven classification tasks. We investigated different strategies to classify the odor data and provided a suitable approach with accuracy of 99% and with inference times under 1.8 seconds. We found that with the techniques used the operation of the e-nose system can differentiate between different brands of whiskey if they have different distillation procedures (such as blended or single malt), but for a better separation between a higher number of different types of whiskies, we need a dataset wide enough to achieve higher accuracy in the separation of whiskies with the same distillation procedure. In the case of clearly different substances, as subproducts of the whiskey distillation process, the procedure established in this work works fine (achieving a high accuracy in the classification process) with an accuracy around 96%.

From the present work, we note that an electronic nose such as the one used is not yet able to replace the olfactory capacity of a good sommelier/nose, but with a dataset good enough we could have this possibility. However, we have made progress and we will continue the work using the developed software framework in other e-nose devices, which may have more channels, and provide a better sensitivity. For future work, we are working on different datasets for whiskey, wine and beer quality classification. Also, datasets for the different stages of the distillation or fermentation process could be helpful for monitoring and forecasting final the quality of the product.

## Data Availability

https://github.com/jausegura/Odor-sensing.
